# Development of a Gastight Thoracotomy Model for Investigation of Carbon Dioxide Field-Flooding Efficacy

**DOI:** 10.7759/cureus.21099

**Published:** 2022-01-11

**Authors:** Mira Puthettu, Stijn Vandenberghe, Stefanos Demertzis

**Affiliations:** 1 Department of Cardiac Surgery, Istituto Cardiocentro Ticino, Lugano, CHE; 2 Faculty of Biomedical Sciences, Università della Svizzera Italiana, Lugano, CHE

**Keywords:** minimally invasive cardiac surgery, gastight model, co2 field flooding, thoracotomy, in-vitro study

## Abstract

Carbon dioxide (CO_2_) field-flooding during cardiac surgery is a prevention technique to avoid blood-air contact and subsequent embolization. Although it was first used more than 60 years ago, there is still some perplexity around its efficacy, mainly because the gas is invisible and air embolization is difficult to quantify.

An accurate assessment of field-flooding can, therefore, best be performed in models where various methods can be tried in a controlled environment and evaluated with industrial-grade sensors. Multiple options are available for anatomically correct models that reproduce a sternotomy situation, but models for minimally invasive cardiac surgery are expensive and normally meant for training of surgical techniques where only the top side of the model is important.

We created a low-cost and “home-made” gastight mini-thoracotomy model with internal organs and left atrial incision to investigate CO_2_ insufflation in a simulated minimally invasive mitral valve surgery. The model was validated with CO_2_ field-flooding tests with a commercial diffuser, while three sensors continuously registered the local concentration of CO_2_ gas.

## Introduction

The human circulatory system is developed as a closed fluid circuit, where blood is shielded within the heart chambers and blood vessels. During open-heart surgery, however, the opening of the circulation exposes blood to air, which results in the formation of air bubbles on the blood surface, especially in the left ventricle and the right upper pulmonary vein [1], which may embolize later with the risk of critical organ damage. Recent studies with pre- and post-operative psychological assessments and improved imaging techniques have found that much more covert brain insults and micro-bleeds are caused by open-heart surgery than originally thought [2]. Despite this fact, many surgeons routinely use a preventive technique called “field-flooding”, which begs the question, is this technique effective [3].

Field-flooding is similar to carbon dioxide (CO_2_) insufflation in visceral surgery, but with a different purpose; it involves filling the surgery site and chest wound with free-flowing inert CO_2_ gas to push away the air and this avoids all blood-air contact and its inherent risks. In a sternotomy procedure, the heavy CO_2_ fills the chest like a bowl and pushes the air upward and out of the chest, eventually forming a dome of CO_2_ where new air supposedly cannot enter. Nonetheless, when performing a minimally invasive cardiac surgery (MICS) via a thoracotomy, the incision is on the side of the patient and the air needs to be forced out via a more complex pathway, which could potentially reduce the efficacy of this prevention technique.

To date, clear evidence on CO_2_ insufflation’s efficacy is still to be found due to several contradictory clinical studies [4,5], likely due to the lack of good outcome variables and variability in gas diffuser types used, their installation in the patient, and the gas flow rates applied. Hence, laboratory setups can be more suitable for quantified comparisons of different techniques, where models of the surgery site can be provided with gas concentration sensors, whose measurements can be used as surrogate outcomes for the patient protection level.

Previous in-vitro studies regarding CO_2_ insufflation are rare and generally performed in sternotomy models by only two research groups, including our own [6-8]. The one MICS model we are aware of was developed by Nyman et al. who tested a novel miniature CO_2_ diffuser [9], but insufficient information was provided to replicate this model. In general, commercial medical training models are no option for CO_2_ studies, because they are not designed to be gastight and are expensive. Thus, we took inspiration from some low-cost and “home-made” alternatives that have been developed, especially for student training in emergency thoracotomy [10-12]. We dedicated this technical report to give a detailed description of a thoracotomy model that is specifically designed for CO_2_ field-flooding studies and can also be used for training other surgical procedures.

## Technical report

Design considerations

Contrary to thoracotomy models used for training of emergency access or surgical skills, where specific anatomic structures and corresponding texture are of key importance, in the case of CO_2_ studies, the priority is on faithful reproduction of internal volumes and avoidance of gas leakage. The total internal volume of the mediastinum should consider dead space that is normally occupied by organs. The model should have one heart, two deflated lungs, an aorta, a vena cava, and a trachea.

The only gas exchange pathway for air and CO_2_ should be the incision wound of thoracotomy, which needs to be of realistic size and location as in real surgery and to enable the usage of real surgical tools and instruments. While the model should be gastight with exception of the incision, ideally it should still be possible to open it to quickly evacuate all gases and reset to a baseline atmospheric condition before starting new measurements.

List of materials

An overview of all materials needed is shown in Table 1. In addition to the listed items, universal glue and hot glue were necessary to build the model.

**Table 1:** List of materials with the corresponding effective price calculated based on needed quantity.

Item	Quantity	Effective cost (USD)
From grocery store:		
Big Tupperware, 4.2 L, airtight lid 21 cm x 15 cm	1	20.00
Sandwich bags, 100 x 3 L	3 x 3 L	0.08
Small Tupperware, 0.75 L, airtight lid 15 cm x 12 cm	1	12.00
From hardware store:		
Flexible electrical cable conduit, M20, l = 10 m	length = 18.5 cm	0.26
Mounting clip	2	6.00
Plug	5	0.38
Rigid electrical cable conduit, l = 2 m, d = 2 cm	length = 25 cm	15.00
Spiral wrap tubing (SW4, AlphaWire), d = ½”, l =7.62 m	length = 5 cm	0.20
T connector	1	3.00
Expired product from hospital:		
Pressure sampling line	1	n/a
Silicone perfusion tube, d = ½”, l = 35 cm	1	n/a
Soft tissue retractor (Ref C8401, S 2.5-6 cm, Alexis O)	1	n/a
Waste collection bag for cell saver, 10 L, Medtronic	1	n/a
Miscellaneous:		
Barbed Luer connector, male, 25 pieces	1	0.96
Barbed Luer connector, female, 25 pieces	1	0.96
Felt sheet		5.00
Heart model (Lake Forest Anatomicals Inc, Lake Forest, IL)	1	75.00
Hollow mannequin torso (Albert Ladenausstattung GmbH, model: BU946280)	1	70.00
Hook and loop adhesive tape		3.00
Life-size skeleton (Anatomy Online, model: AO SM 100, 180 cm tall)	1	182.00
Total		393.84

Steps to build the model

Step 1: Heart and Large Vessels

**Figure 1:**
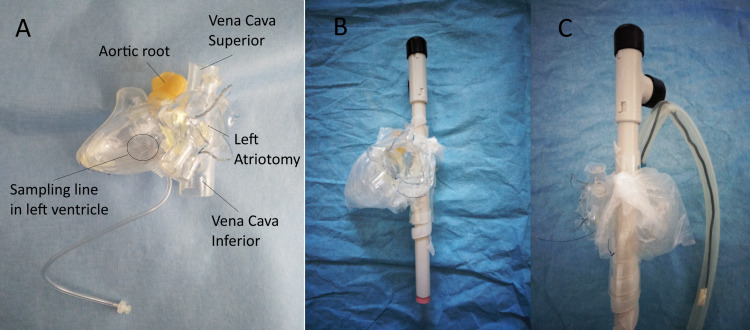
Step 1 - heart and large vessels. A: Heart model with sampling line in the left ventricle. B: Heart wrapped in sandwich bag (pericardium) and pipe tube with T connector inserted to simulate vena cava. C: Introduction of silicone tube to reproduce aorta.

The heart model was first modified by drilling a 4 mm hole in the anterior wall of the left ventricle (LV) and pressure line tubing was inserted to form a gas sampling line that sits flush with the internal roof of the LV. An H-shaped incision was made in the left atrium and the resulting petals were folded open while making use of a heat gun to form a hole simulating a typical opened and retracted mitral valve approach. The final modified model is shown in Figure 1A.

The heart model was then wrapped inside a sandwich bag (pericardium) with holes for the main vessels. A rigid electrical cable conduit (light grey) was inserted through the vena cava (superior and inferior) to extend it (Figure 1B). A T connector with sealing plugs (black) was added to the pipe to simulate bifurcation in left and right brachiocephalic veins and avoid gas intrusion, respectively. The spiral wrap was used to compensate for the diameter mismatch between vessels and pipe and to obtain a tight fit. The aorta was simulated with a 1/2’’ silicone tube containing a malleable solid strand electrical wire to replicate the shape of the aortic arch (Figure 1C). The remaining open ends of pipes and tubes were sealed gastight with silicone plugs (pink and blue).

Step 2: Heart Positioned Inside the Thoracic Cavity

First, the top corners of a waste collection bag from a set of cell saver disposables were heat sealed to reduce internal volume and simulate the tapering of the upper chest. Ribbed flexible electrical cable conduit (blue) was glued at the top of the taper and ran inside the bag to simulate the trachea, with both ends sealed.

**Figure 2:**
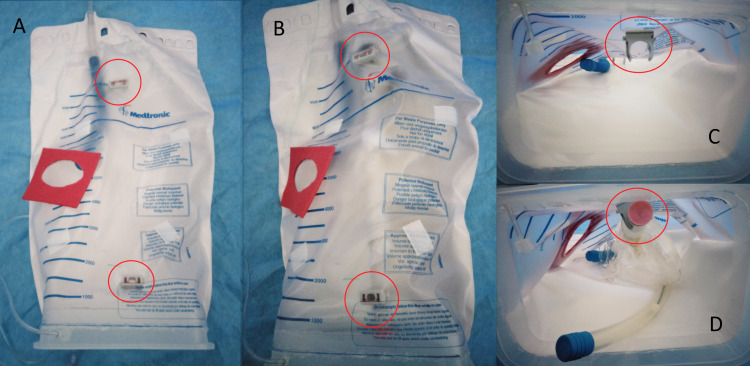
Step 2 - heart positioned inside the thoracic cavity. A: Thoracic cavity with only trachea. B: Insertion of heart model and large vessels. C: View inside of the thoracic cavity with only trachea. D: View inside of the thoracic cavity with heart model and large vessels. Pipe clips to keep the heart in position are shown by red circles in all figures.

**Figure 3:**
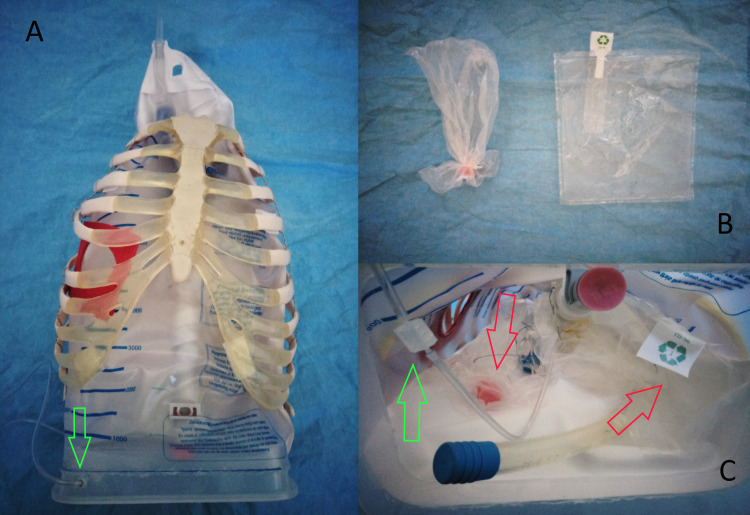
Step 3 - rib cage with organs. A: Waste collection bag inserted in the rib cage and attached by hook and loop tape (green arrow shows the position of the gas sampling line). B: Sandwich bags were used to simulate deflated lungs. C: Simulated organs inside the thoracic cavity (green arrow shows gas sampling line, red arrows show the position of lungs).

The bottom 5 cm of the waste collection bag was trimmed off to achieve access to the inside while a large Tupperware container was split such that the top 5 cm and the lid remained. This rectangular Tupperware "band" was glued inside the trimmed edge of the bag to achieve a gastight flexible chamber with a removable lid to simulate the mediastinum (Figure 2A). A hole was made through this Tupperware "band" to feed through a pressure line tubing, which was attached to the previous one in the LV via barbed Luer connectors (see green arrows, Figures 3A, 3C). This is later used to connect a gas sampling line from the LV to an external CO_2_ concentration sensor without compromising the gas tightness of the model.

A hole to match the left atrial incision was made in the waste collection bag to reproduce a surgical incision wound (length of 8.5 cm, width of 4 cm). The wound circumference was reinforced with a piece of felt with a matching hole (Figures 2A, 2B). Two mounting clips for the electrical conduit were glued to the internal anterior wall of the waste collection bag to fix the vena cava conduit and install the heart model in a reproducible position (see red circles in Figure 2).

Step 3: Rib Cage With Organs

A rib cage with all thoracic vertebrae was scavenged from a plastic skeleton model. The waste collection bag with heart and vessels was placed inside the rib cage and the anterior wall was suspended with four hook and loop attachments (white rectangles in Figure 2B) whose counterparts were glued on the internal sides of the 4th and 10th ribs. The final organs to be added were the deflated lungs, which were simulated by sealed-off, crumbled sandwich bags (Figure 3B) that were inserted below and around the heart, high up in the waste collection bag (Figure 3C).

Step 4: Gastight MICS Model

**Figure 4:**
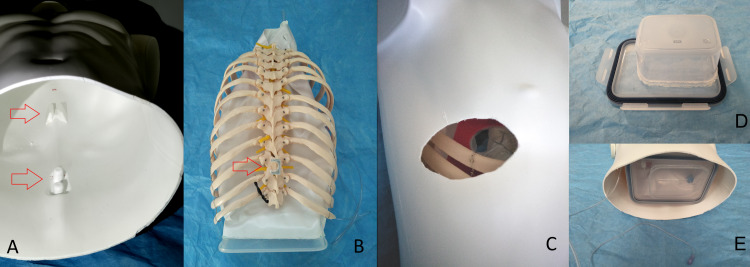
Step 4 - gastight MICS model. A: Mannequin torso with mounting points (shown by red arrows) on the internal wall. B: Clip (shown by red arrow) on the back of the rib cage. C: Rib cage inserted in the mannequin until incision holes match. D: Small Tupperware’s container glued on the lid of big Tupperware’s container to simulate diaphragm. E: Thoracic cavity closed off with lid to be gastight.

To install the ribcage with mediastinum and organs consistently inside the mannequin torso, the existing mounting bosses on the inner posterior side of the mannequin (bottom red arrow in Figure 4A) were used and a matching three-dimensionally (3D) printed clip (red arrow in Figure 4B) was glued on the T10 vertebra. An additional Y-shaped channel was 3D printed and glued more cranially inside the torso as a guide rail for the first vertebrae (top red arrow in Figure 4A). A hole was cut in the right thoracic wall of the torso model to match the incision wounds of the mediastinum and left atrium (Figure 4C).

The small Tupperware container was hot glued on the inside of the large Tupperware’s lid to simulate the concave diaphragm and to obtain a realistic mediastinum volume (Figure 4D). After all components inside the torso were arranged to align the incisions, the bottom of the waste collection bag was closed with the lid (Figure 4E) and a soft tissue retractor was inserted in the incision wound to form a sealed tunnel between the external atmosphere and the gastight mediastinum, thereby preventing gas to flow in the free spaces between the torso and rib cage.

Testing CO_2_ insufflation

To test the functionality of the model for CO_2_ field-flooding studies, CO_2_ gas was delivered inside the mediastinum via a CarbonMini diffuser (Cardia Innovation AB, Kungens Kurva, Sweden) with a flow rate of 5 L/min. The diffuser was installed as in common MICS: inserted via the main incision (through the soft tissue retractor) and curved upward towards the anterior wall.

CO_2_ was measured at three different sampling spots: in the LV, next to the diaphragm at the posterior wall, and vertically below the tip of the diffuser at the posterior wall. In each spot, a sampling line was installed and connected to a membrane pump, which continuously lead the sampled gas at 250 ml/min over calibrated CO_2_ sensors (SprintIR-6S, GasSensingSolutions, Cumbernauld, UK). All raw concentrations from sensors were recorded in real-time by Matlab (Matlab 2017b). Delivery CO_2_ flow was monitored by a digital flowmeter (MassView, Bronkhorst High-Tech B.V., Ruurlo, Netherlands).

**Figure 5:**
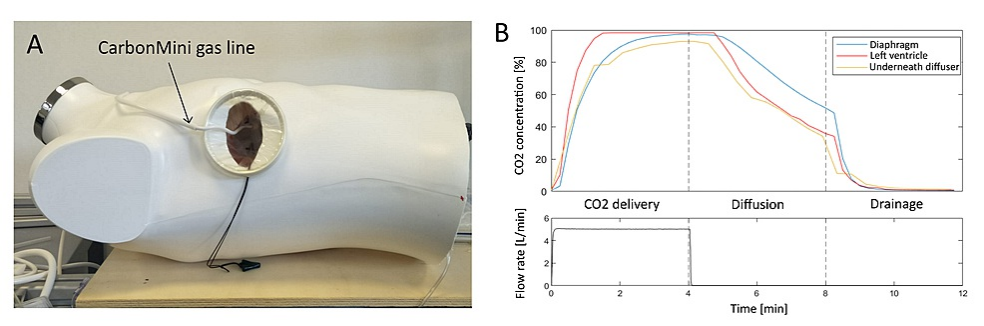
Final mini-thoracotomy model for minimally invasive cardiac surgery (MICS) simulation. A: Picture of the final model with CarbonMini positioned for CO_2_ insufflation. B: CO_2_ concentrations during the three phases of the recording: CO_2_ delivery at 5 L/min, diffusion (lid on), and drainage (lid off).

The data recording displayed in Figure 5 is divided into three phases of four minutes each: (1) CO_2_ insufflation at 5 L/min was started and kept constant, (2) CO_2_ insufflation stopped and diffusion from the closed model was monitored (lid on), and (3) the lid was removed to drain the gas from the model. Figure 5 shows CO_2_ concentrations obtained during insufflation via CarbonMini. The highest CO_2_ values were obtained in the LV, reaching 100% after two minutes. During diffusion with the lid on, all sampling spots showed a gradual decrease in CO_2_ concentrations. A decrease of 65% was observed in the LV, and of 55% underneath the diffuser. It was slower next to the diaphragm, which was the farthest place from the incision wound. At this sampling spot, CO_2_ concentration was 50% after four minutes from the insufflation stop. Once the lid was removed, CO_2_ concentrations dropped to less than 1% in two minutes.

## Discussion

The current model was designed and built specifically for CO_2_ studies, taking inspiration from emergency thoracotomy models by focusing on internal volume and gas tightness. Thanks to the holes in the left ventricle and the Tupperware’s "sealing band," as well as the left atriotomy, gas sampling line tubing can be easily positioned where preferred and more insights on the distribution of CO_2_ concentration inside the thoracic cavity can be obtained.

The test using a CarbonMini diffuser to blow CO_2_ showed that a concentration of 100% was reached inside the left ventricle and was kept stable until insufflation stopped, indicating that the surgical field was sufficiently flooded. Diffusion after stoppage was fast and shows that a continuous insufflation is necessary in real surgery, but for measurement protocols on the model, a full reset of the CO_2_ concentration was achieved most efficiently by removing the lid, which caused rapid drainage of the heavy gas out of the model. This is a useful feature for speeding up comparative testing of multiple conditions, where the initial situation (no gas in the thorax) should be identical. Moreover, no practical inconvenience was encountered during this validation test. All these aspects of the model result in a good testing environment to evaluate CO_2_ field-flooding in a controlled way.

This MICS model has several advantages in addition to the gastight thoracic cavity. The current model is anatomically far more detailed than our previous sternotomy model [8] and it is unique as no descriptions were found about similar models in the literature. It is re-usable and contains dismountable components, which means that its mediastinum content can be modified and adapted to the user, while the external appearance is fixed (mannequin torso). So, the model could be high-fidelity or could be simplified too. The heart model can also be easily switched with an animal heart for the training of new cardiac surgeons or new procedures or devices. Since the incision has a realistic size, it can be used to practice complex procedures, such as the introduction of valve devices or correct positioning of the atrial retractor. A skin layer can be added to the external wall of the torso to practice incision and retractor placement. The addition of extra surgical instrumentation, such as a trocar, is also possible by simply drilling a new hole in the mannequin, but in this case, gas tightness should be guaranteed in a different way. Even fancier upgrades are an option: the model can be used as starting point for augmented reality, which can be useful for training or a good learning experience.

The here suggested MICS model is much cheaper than professional models, but due to the use of a commercial torso and rib cage, it is rather expensive compared to other “home-made” alternatives that work solely with items found in a hardware store. For our purposes, the value increase is certainly worth the extra investment. Nonetheless, this article simply presents detailed guidelines for one possible MICS model, so these instructions can be the kick-off to unleash the readers' creativity.

## Conclusions

As long as a reliable measurement of in situ carbon dioxide concentration is not possible in the thoracic cavity during surgery, more standardized and high-fidelity models are necessary to find clear evidence on CO_2_ field flooding’s efficacy. Due to the lack of minimally invasive cardiac surgery models that are gastight, we provide here a “home-made” alternative for gas-related experiments.

Although the suggested model is intended for CO_2_ studies, it can be modified and used for other purposes. Its components can be easily dismounted and replaced according to users’ needs, so it can be a good option for training and learning.
